# Quercetin-Based Nanocomposites as a Tool to Improve Dental Disease Management

**DOI:** 10.3390/biomedicines8110504

**Published:** 2020-11-16

**Authors:** Giuseppe Angellotti, Denise Murgia, Giuseppina Campisi, Viviana De Caro

**Affiliations:** 1 Dipartimento di Discipline Chirurgiche, Oncologiche e Stomatologiche, Università degli Studi di Palermo, 90127 Palermo, Italy; giuseppe.angellotti@unipa.it (G.A.); denise.murgia@unipa.it (D.M.); giuseppina.campisi@unipa.it (G.C.); 2 Dipartimento di Scienze e Tecnologie Biologiche Chimiche e Farmaceutiche (STEBICEF), Università degli Studi di Palermo, 90123 Palermo, Italy

**Keywords:** quercetin, antibacterial effect, dental disease, dental implant, osteoinductive effect, quercetin scaffold

## Abstract

The restoration and prosthetic rehabilitation of missing teeth are commonly performed using dental implants, which are extremely effective and long-lasting techniques due to their osteointegration ability with the preimplant tissues. Quercetin is a phytoestrogen-like flavonoid well known for its several positive effects on human health, mostly linked to the anti-inflammatory, antioxidant, and antibacterial activities against both Gram-positive and Gram-negative bacteria. Moreover, many studies in dentistry and the maxillofacial fields have highlighted the positive effects of quercetin on osteogenesis, acting on osteoblast activity and angiogenetic process, and promoting soft and hard tissue regeneration. This review focuses on the role of quercetin on the healing and restoration of bony defects, considering the experimental findings of its application both in vitro and in vivo as a mere compound or in association with scaffolds and dental implants having functionalized surfaces.

## 1. Introduction

**Figure 1:**
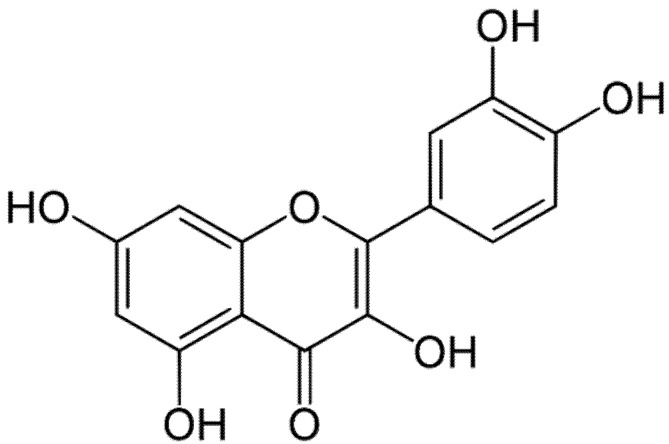
Chemical structure of quercetin (QRC).

Dental implants are widely used in modern dentistry as a revolutionary way to replace missing teeth due to their capability of integrating with the surrounding tissue, commonly called peri-implant tissue. A close contact between the implant and the peri-implant bone is fundamental to achieve optimal osseointegration, which is considered a necessary condition for loading and long-term clinical success of the dental implants [1]. All the implants on the worldwide market are constituted of bioinert materials, and among these the most used are titanium (pure or as an alloy), ceramics, bio-glasses, and bio-composites [2]. The use of dental implants has a high success rate even if some cases of failure have been reported. Several factors are involved in implant failure, as, for example, an insufficient osseointegration related to poor bone quality or peri-implantitis, which lead to complications in the peri-implant tissue [3]. Peri-implantitis is caused by a massive inflammatory response of soft tissue against bacterial infections and the biofilm formation around the surface of the implants, which can also affect the hard tissue, resulting in osteolysis and bone mass loss [4]. The microorganisms involved in the onset of this disease are predominantly Gram-negative bacteria, such as *Aggregatibacter actinomycetemcomitans*, *Porphyromonas gingivalis*, *Prevotella intermedia*, *Tannerella forsythia*, and *Treponema denticola,* but it has been shown that Gram-positive bacteria, such as *Staphylococcus aureus* and enterococci, can also colonize and develop biofilms on an implant’s surface [5]. In this respect, dentistry is focusing its attention on the application of polyphenols as a coadjutant of the healing processes after implant loading [6,7]. Polyphenols are a wide group of biologically active compounds present in plant-based foods. Based on the characteristics of the aromatic ring and functional groups, they could be subdivided into four main classes: flavonoids, lignins, phenolic acids, and stilbenes [8]. Nowadays, the interest of scientific communities in these compounds is still increasing due to their beneficial effects in human health, as well as their antibacterial and wound-healing properties [9,10]. Polyphenols, despite having a wide range of positive activities, suffer from poor bioavailability, which limits their effectiveness after oral intake. To overcome this problem, many efforts have been proposed, such as the entrapment of polyphenols in drug delivery systems (DDSs) or scaffolds, which could represent a valuable resource for local and topical treatment of periodontal structures and mucous membranes [11]. Quercetin (QRC) is a flavonoid belonging to a group of plant-derived nonsteroidal compounds called phytoestrogens. It is a polyphenol-based molecule (Figure 1) and it is widely distributed in many vegetables, fruits, seed, grains, and also in tea and wine [12].

In recent years, the focus on this compound has increased due to its several therapeutic properties, such as anti-inflammatory [13,14,15], antioxidant [16], anti-tumoral [17], anti-ulcer [18], anti-allergy [19], anti-diabetic [20], antihypertensive [21], immunomodulatory [22], and gastroprotective [23] activities. Moreover, the proven antiviral [24] activity has suggested its administration in the prevention and treatment of SARS-CoV-2-related disease [25]. It also possesses a significant antibacterial effect against Gram-positive and Gram-negative bacteria [26]. Despite its beneficial effects, QRC suffers from low bioavailability after oral administration [27] and degrades rapidly when exposed to light and in alkaline aqueous solutions [28,29]. In animal studies and human trials, it has been shown that only a small amount of dietary QRC, approximately 6%, was adsorbed by the gastro-intestinal (GI) tract [30]. On the one hand, this phenomenon is due to QRC’s low solubility in GI fluids [30], given that the more soluble QRC-glycoside derivatives (conjugated with sugars as rhamnose, glucose, etc.) from plants undergo hydrolysis already in the oral cavity [31]; on the other hand, in the stomach the strong acid environment degrades the QRC structure into phenolic acids [32]. To overcome these drawbacks, novel formulations based on micelles and nanoparticles have been proposed to increase the oral QRC bioavailability and improve its stability [33,34]. Therefore, in recent years, the advantage of its administration by topical formulations has been highlighted, principally due to its antioxidant, anti-inflammatory, and antibacterial activities [35].

## 2. The Antibacterial Effect of Quercetin

**Figure 2:**
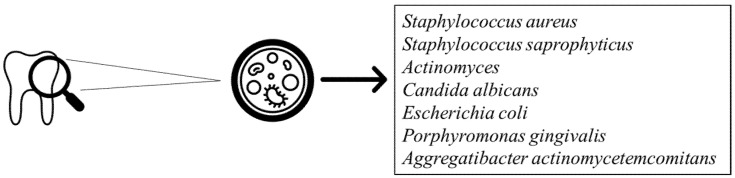
Bacterial strains involved in periodontal disease.

Oral bacterial infections are strongly associated with the most common oral diseases, such as periodontitis and gingivitis. The kinds of bacteria involved are both Gram-positive, including *Streptococcus* and *Actinomyces*, and Gram-negative (Figure 2) [36]. On the one hand, the increase in bacterial number is related to a reduction of innate and adaptative immunity; on the other hand, the growth of drug-resistant bacterial strains makes the treatment of these diseases a challenge. In light of this, the usage of flavonoids, such as QRC, has been proposed as an alternative to common antibacterial treatments [37].

Some studies have investigated the role of flavonoids in the management of dental plaque bacterial infection. Notably, Gutierrez-Venegas et al. have demonstrated that QRC and other flavonoids possessed bacteriostatic activity on almost all microorganisms affecting the oral cavity, including *Candida albicans*. The latter is well known to cause candidiasis, which could manifest in different clinical forms and involves one or more mouth sites [38].

The properties of some microorganism to produce biofilm as well as the drug resistance are contemplated as global public health problems. In particular, biofilm promotes the growth of microorganisms, protecting them from the surrounding environment, acting as an important factor of virulence, and increasing bacterial resistance to antibiotics and the host’s immune system [39]. In a study proposed by Dias da Costa Júnior et al., the antibacterial and antibiofilm activity of QRC against *Staphylococcus aureus* and *Staphylococcus saprophyticus,* both vancomycin and methicillin resistant, was evaluated. QRC displayed the MIC values, ranging between 250 to 1000 µg/mL and 62.5 to 1000 µg/mL for *Staphylococcus aureus* and *Staphylococcus saprophyticus*, respectively. The best inhibitory effect of QRC was observed against the methicillin-sensitive *Staphylococcus aureus* (MSSA), methicillin-resistant *Staphylococcus aureus* (MRSA), and vancomycin-intermediate *Staphylococcus aureus* (VISA) strains (MIC = 250, 500 and 125 µg/mL, respectively), while the lowest inhibitory effect was against the vancomycin-resistant *Staphylococcus aureus* (VRSA) strain (MIC = 1000 µg/mL). Regarding the antibiofilm activity, QRC was able to reduce 50% of the biofilm production, even when it was analysed in sub inhibitory concentrations [40].

An antibacterial mechanism of QRC has been proposed by Wang et al. Firstly, in this study they found that the MICs of QRC for *Escherichia coli* and *Staphylococcus aureus* were 0.0082 and 0.0068 µmol/mL, respectively. After that, both bacterial strains were treated with concentrations of QRC up to 500-fold higher than their respective MICs for 24 h and then analysed by transmission electron microscopy (TEM) and in term of alkaline phosphatase (ALP) extracellular activity, to investigate the morphology and the permeability of the bacteria. The TEM images displayed that the cell wall and membrane were already damaged using concentrations 50-fold higher than the MIC for *Escherichia coli,* and 10-fold higher for *Staphylococcus aureus*. At the same conditions, the researchers found that the ALP activity in *Staphylococcus aureus* was significantly higher than *Escherichia coli*, confirming the previous results. In conclusion, according to this study, the bacteriostatic effect of QRC is greater for Gram-positive than Gram-negative bacteria, probably related to the difference in peptidoglycan concentration in the cell wall [41].

Since *Porphyromonas gingivalis* (Pg) and *Aggregatibacter actinomycetemcomitans* (Aa) are both strongly associated with the onset of periodontitis, Geoghegan et al. have studied the effect of QRC against these bacteria. Both the in vitro cultures of these periodontal pathogens were treated with an aqueous QRC solution of 0.1, 0.05, and 0.025 g/mL, using chlorhexidine and sodium chloride as a positive and negative control, respectively. All the results were carried out after 1, 3, 6, and 24 h of incubation. They found that QRC was able to reduce significantly the growth of Pg at 1 h and no difference with the positive control after 6 h was detected, while the growth of Aa was inhibited in a time-dependent manner when compared with the negative control. Finally, in another study they showed that QRC possesses an antibacterial activity of 0.0125 g/mL and 0.1 g/mL MIC for Pg and Aa, respectively [42].

## 3. Effect of Quercetin on Bone Tissue Regeneration

Phytoestrogens are plant-derived dietary chemicals found in a lot of vegetables and fruits consisting of isoflavonoids, lignans, stilbenes, and the flavonoid QRC [43]. Some studies have displayed their role in prevention and treatment of cardiovascular diseases, osteoporosis, diabetes, obesity, and menopausal symptoms [44,45]. They are chemically characterized by structural similarity to 17-β-oestradiol (E2), which enables them to modulate the osteogenic mechanism by interaction with ERa and ERb, subtypes of the oestrogen receptors (ERs), both present on the surface of osteoblasts cells [46]. The effect of QRC on osteogenesis has been confirmed by several studies [47,48] and it is considered the most potent osteogenic chemical ever discovered, although the real mechanism involved is still unclear.

Pang et al. have investigated the effect of QRC in the proliferation and osteoblastic differentiation on the in vitro cultures of mice-derived bone marrow mesenchymal stem cells (BMSCs). The latter are multipotent stromal cells able to differentiate into various cell types, including the osteoblasts, and therefore play an important role in the induction of osteogenesis [49]. After treatment with increasing concentrations of QRC (0.1 μM–5 μM), they observed a significant rise in BMSC proliferation and ALP activity, which meant an enhanced osteoblast formation in a dose-dependent manner. They also displayed that QRC stimulated osteogenic differentiation, interacting with the oestrogen-signalling pathway, which led to the up-regulation of the osteogenic genes runt-related transcription factor 2 (RUNX2) and Osterix (OXS) [50].

The stimulatory effect of QRC on ALP activity was evaluated by Prouillet et al. In one study, they worked on human osteoblastic cell MG-63 incubated with QRC at 1, 10, and 50 μM for 24 and 48 h in Dulbecco’s modified eagle medium (DMEM), observing that QRC was able to rapidly increase the ALP activity in time and dose-dependent manners. They also found that the extracellular signal-regulated kinase (ERK) pathway was involved in the mechanism, highlighted by the reduction in QRC’s effect when the cells were treated with the MEK inhibitor PD 98059. They evidenced that ICI 182780, an antagonist of the oestrogen receptors (ERs), was also able to reduce the quercetin-mediated ALP activity, confirming the role of these receptors in the phenomenon [51].

Since is well known that the mitogen-activated protein kinase (MAPK) pathway was involved in the osteogenic differentiation of mesenchymal stem cells (MSCs), Li et al. have evaluated the role of QRC in this phenomenon. The MCSs obtained by mice were treated with QRC at 0.01, 0.1, 1, 10, and 100 μM for 72 h. After incubation, the authors found that QRC stimulated the ALP activity in a dose-dependent manner and increased the levels of osteocalcin (BGP) and type 1 collagen (COL I), two bone marker proteins. Moreover, the MAPK and ERK pathways were also stimulated by the treatment with QRC; these results were confirmed, incubating the cells simultaneously with QRC and antagonists of these pathways. The authors highlighted that all the above-mentioned markers were downregulated, confirming the role of QRC in the osteogenesis of MAPK [52].

QRC seems to influence also the osteoclastogenesis phenomenon. In a study carried out by Wattel et al., the in vitro role of QRC on osteoclastic differentiation was evaluated using two major models of osteoclastic-like cells, RAW 264.7 and PBMC, treated with various concentrations of this flavonoid up to 10 μM. They found that QRC showed a strong inhibitory effect in osteoclast differentiation at concentrations as low as 1 μM. Their investigations highlighted that QRC inhibits two transcription factors, the nuclear factor kappa-light-chain-enhancer of activated B cells (NFkB) and the activator protein 1 (AP-1), which are well known to modulate the differentiation and proliferation of osteoclasts [53].

A summary of QRC’s antibacterial activity and bone tissue regeneration properties is provided in Table 1.

## 4. Dentistry Application

In the last years, some studies have highlighted the effects of QRC in oral and dentistry applications. Researchers have demonstrated that both the osteogenesis and anti-inflammatory properties of QRC could be used to manage the dental restoration and periodontitis.

Since the stimulation of dentinogenic differentiation could accelerate pulp reparation after pulp-capping treatment, Kim et al. have investigated the role of QRC, genistein, and baicalin in this phenomenon in terms of ALP activity, dentin sialophosphoprotein (DSPP) mRNA expression, and mineral deposition. Increasing concentrations from 1 to 25 μM of QRC and other chemicals were tested on human dental pulp cells (HDP) culture that had been isolated from orthodontic patients’ incisors. Among all, QRC showed the highest ALP activity and DSPP mRNA expression. As the latter is an odontoblastic-specific protein, this result suggested a dentinogenic activity of QRC. Therefore, they also found that the cells treated with QRC displayed an increasing mineral deposition in a dose-dependent manner. Finally, the quercetin-related ERs and ERK signalling pathway have been hypothesized as having a role in dentinogenesis, but the results have not confirmed it yet [54].

The role of QRC as a remineralization agent on root caries treatment was evaluated by Epasinghe et al. For this purpose, demineralized root fragments obtained from human third molars were treated with a solution of 6.5% w/v QRC in phosphate buffer for 10 min. In the same way, a sodium fluoride solution and deionized water were used as the positive and negative control, respectively. All samples were treated cyclically six times per day with QRC solutions, an acidic buffer, and a neutral buffer for 8 days. Remineralization was assessed by transverse microradiography and confocal laser scanning microscopy. According to the results, QRC was able to inhibit the demineralization. Therefore, it promoted the remineralization of root caries lesions more than the negative control, although fluoride remained the most effective [55].

A reduction in the dentin-adhesive bond strength could lead to failure of the dental restoration due to the increasing metalloproteinases (MMPs) activity and acidogenic bacteria proliferation, such as *Streptococcus mutans*. A composite dental adhesive doped with QRC at various concentrations was developed by Yang et al. and evaluated in vitro and in situ. They found that the quercetin-based composite reduced both *Streptococcus mutans* biofilm growth and metabolic activity in a dose-dependent manner up to a QRC concentration of 1000 µg/mL. The composite was characterized in terms of microtensile bond strength, nano leakage expression, and MMPs activity by in situ analysis on caries-free human third molar specimens. The results highlighted that QRC exhibited no influence on the immediate bond strength at a range concentration of 100 to 500 µg/mL. Therefore, both nano leakage expression and MMPs activity decreased with the rising concentration of QRC. The overall results suggested that the adhesive loaded with QRC was more efficient than the empty one to preserve the degradation of the adhesive–dentin interface [56].

Napimoga et al. have evaluated the role of QRC on a mouse periodontitis model infected with *Aggregatibacter actinomycetemcomitans*. After three days of bacterial inoculation, to induce the periodontitis, the mice were treated with a QRC daily dose of 100 mg/kg, for 15 days, by subcutaneous injection. Then, on the sacrificed animals, the morphometric analysis highlighted that QRC reduced the alveolar bone resorption while it did not affect the viability of the bacteria colonies. An immunohistochemical assay conducted on gingival tissue has shown that QRC negatively modulated the production of pro-inflammatory cytokines, such as the interleukin-1β (IL-1β), tumour necrosis factor-α (TNF-α), and interleukin-17 (IL-17). Moreover, QRC downregulated the intercellular adhesion molecules I-CAM and osteoclastogenic receptor activator of nuclear factor kappa-Β ligand (RANKL) expression. These results suggested that QRC could be useful in the treatment of *Aggregatibacter actinomycetemcomitans*-related bone loss [57].

## 5. Quercetin Formulations for In Situ Osteogenesis

In the light of evidence about QRC’s strong effect in promoting osteogenesis, different strategies have been proposed to overcome its low bioavailability after oral administration; in particular, the development of targeted, sustained-release systems as scaffolds or drug delivery systems (DDSs) that are able to improve the QRC effects locally on bone tissue regeneration.

According to Wong et al., a collagen matrix mixed with QRC locally increases the bone formation when grafted into a skull defect. This study was assessed on 5-month-old New Zealand white rabbits with six defects in each parietal bone. They observed that the defects treated with QRC in the collagen matrix presented a total of 556% more new bone than those treated with the collagen matrix alone. These results confirmed the role of QRC in osteogenesis and bone healing [58].

Hydroxyapatite bioceramic microspheres with a nano-micro hybrid surface, loaded with QRC at a concentration of 200 μM (nHA/Quer) and without QRC (nHA), were prepared by Zhou et al. for the management of osteoporosis. QRC was released by the microspheres in a sustained manner for 28 days. Two groups of ovariectomized rats (OVX), both presenting inducted monocortical plug bone defects, were randomly treated with nHA or nHA/Quer particles, directly implanted into the injured sites. After eight weeks of implantation, the group treated with nHa/Quer, analysed by microfilm perfusion and micro-CT, showed a more massive formation of new bone mass and higher vessel area compared with the ones treated with nHA, demonstrating that QRC possesses osteoinductive activity and angiogenetic effect [59].

3D printing and thermally induced phase separation technology have been used by Zhu et al. to develop an innovative composite scaffold constituted of porous poly (L-lactide)/chitosan functionalized with polydopamine and QRC at a 200 μM concentration. The scaffold was analysed in vitro both on MC3T3-E1 and RAW 264.7 cells cultured for 7/14 days and 24/48 h, respectively. The results showed that the scaffold displayed an optimal osteogenic activity in terms of calcium deposition, ALP activity, and osteogenic related-genes Runx-2, COL-I, and OCN expression. Moreover, it is able to counteract the inflammatory response by a reduction in TNF-α and interleukin-6 (IL-6) expression [60].

An innovative in vitro triculture model involving osteoblast (OB), osteoclast (OC), and endothelial cells (ED) was used by Forte et al. to investigate the osteogenesis effects of a hydroxyapatite scaffold functionalized with increasing amounts of QRC up to 3.1 wt% for 7 and 14 days. They found that QRC in a time- and dose-dependent manner positively influenced the bone microenvironment. Monitoring ALP, COLL1, and the osteoprotegerin (OPG)/RANKL ratio, the authors confirmed that QRC enables osteogenesis by stimulating the osteoblast activity and proliferation, downregulating the osteoclast differentiation and promoting the angiogenetic process necessary for new bone formation [61].

## 6. Scaffold and Biomaterial for Implant Application

The use of dental implants in maxillofacial surgery has improved the standard of living of patients affected by edentulism, mainly due to the type of biomaterial of which they consist. In order to improve the persistence of implants for years, the materials must be biocompatible and bioinert, allowing the complete compatibility between the dental implant and surrounding tissues. Titanium (Ti) pure and its alloys are the most used biomaterials in implantology. This is related to its chemical and biological properties, as well as its ability to form oxide derivatives on the surface, which confer mechanical strength and corrosion resistance [62]. Ti is usually considered a biocompatible material, although some clinical reports, in rare circumstances, have shown an allergic reaction in the patients receving Ti implants [63]. Recently, for aesthetic purposes, implants based on ceramics as biomaterials have been proposed. In particular, zirconia oxide (ZrO_2_) has excellent mechanical properties that make it suitable for dental implants [64]. Therefore, ZrO_2_ is more biocompatible than Ti with comparable osseointegration properties. However, it is not a very versatile material in implantology due to its fragility and its aptitude to fracture [65]. In 2009, a new biomaterial named Roxolid^©^ composed of Ti and Zr was developed. It possesses better characteristics than pure Ti, such as stronger mechanical properties and greater biocompatibility [63], also attributed to the absence of aluminium in its composition [66]. Furthermore, Zr seems able to improve the osteoblast adhesion allowing quicker osseointegration [67]. Nowadays, the researchers have moved their attention to the development of dental implants based on polymers, as they are materials with excellent mechanical and biological properties. Moreover, emerging 3D-printing techniques allow for a quick and accurate realization of the prosthesis and scaffold-based polymer, opening new frontiers in dental implant evolution [68].

Regarding the osteoinductive and antibacterial effects, QRC has been proposed as a key molecule preventing the failure of implants and facilitating the healing process.

A drug-releasing implant (DRI) constituted of nanotubes of titanium dioxide (TNT) covered with chitosan and loaded with QRC was designed by Mohan et al. Four groups of DRI with 0.5%, 1%, 2% weight/weight, and without chitosan, each having 10 mg of QRC, were developed and characterized in term of drug release and surface morphology. The images obtained by the FESEM microscope displayed that the samples were completely covered with polymer and the diameter and height of the nanotubes decreased with the thickness and progressive filling of chitosan. Drug release profiles from the samples were studied in Hanks’ solution for 8 days and the overall QRC released was strongly related to polymer percentage, wherein the TNTs with 0.5%, 1%, 2% chitosan, and ones without chitosan were able to release 50%, 42%, 32%, and 71% of the QRC, respectively. These results showed that by controlling the thickness of the chitosan, the QRC release could be managed and tuned to fit into an optimal therapeutic window for the treatment of post-operative issues and quick bone healing [69].

Catauro et al. have synthesized an innovative biomaterial consisting of inorganic silica matrix and poly-ε-caprolactone (PCL) grafted with QRC to reduce the implant failure related to oxidative stress during the inflammatory response. The influence of various concentrations of QRC and polymer on in vitro bioactivity as well as antioxidant and cytotoxicity activities were evaluated. After 7, 14, and 21 days of soaking in simulated body fluid (SBF), according to the Kokubo procedure [70], the results showed that all the samples were covered on their surface by typical globules of biominerals, concluding that neither the QRC nor PCL concentrations could influence the hydroxyl-apatite crystal formation. The results emerging from the DPPH and ABTS assays highlighted that the antioxidant activity is strongly dependent on the QRC concentration. Therefore, low percentages of PLC were able to increase the QRC scavenger activity; however, high percentages reduced it. Finally, the MTT cytotoxicity assay showed that all the samples were able to reduce the dehydrogenases activity to less than 25%, considering it as an acceptable value for alloys and biomaterials [71].

Titanium surface modification with antibacterial, anti-inflammatory, and tissue-regenerative agents could improve soft tissue regeneration and reduce dental implant failure after maxillofacial surgery. It is well known that the main causes of dental implant failure are related to bacterial infections and massive inflammatory responses that lead to weak soft tissue wound healing around the teeth [72]. According to this proposal, Gomez-Florit et al. have evaluated the in vitro antibacterial and tissue regenerative properties of a titanium surface nanocoated with quercitrin (QUE), the rhamnosilated form of QRC, owing greater bioavailability than QRC due to the presence of the rhamnose. QUE’s positive effects on the regeneration of periodontal hard and soft tissues were also investigated [73]. The titanium surface was previously aminosilanized and then functionalized with QUE by the formation of a covalent bond between the carbonyl group of the flavonoid and terminal amine group of the organosilane. The obtained compound (Ti-Que) was analysed at a 1 mM concentration. They found that the Ti-Que was able to reduce the adhesion and slightly the biofilm formation of *Staphylococcus mutants* compared to the uncoated Ti sample. Moreover, results obtained by human gingival fibroblasts (hGF) cell cultures treated with Ti-Que showed an increase in collagen mRNA production and a reduction in mRNA cyclooxygenase type 2 (COX2) expression that was related to inflammatory processes [74]. In another study carried out by Còrdoba et al., the Ti-Que effects on osteoclastogenesis and osseointegration both in vitro and in vivo were investigated. They highlighted that the Ti-Que implant possessed an osteoclastogenic activity, downregulating the osteoclastic markers (Ctsk,H^+^ATPase, Mmp9) expression on a RAW264.7 cell culture treated with RANKL for 1 week. This result was confirmed by in vivo studies on female New Zealand white rabbits having the implants placed in each tibia for a period of 8 weeks. They observed a significant reduction in the RANKL and osteoclastic marker expression in the animal group treated with Ti-Que, rather than the ones with the uncoated Ti control, while no notable difference between the groups was found, either for ALP or lactate dehydrogenase (LDH) activities. As the presence of LDH and ALP activities in wound fluids are related to the marker of tissue necrosis and the negative index of implant osseointegration, respectively, the latter result suggests that the Ti-Que could reduce osteoclast activity without hindering osteoclastogenesis and bone formation [75]. These results suggested that the enrichment of an implant surface with QRC and QUE could improve peri-implant wound healing and reduce the peri-implantitis onset. A summary of both the in vitro and in vivo QRC-based formulations’ activities on periodontal tissue is presented in Table 2.

## 7. Conclusions

The management of peri-implantitis is fundamental to extend the life of a dental implant and promotes the regenerative processes of dental bone tissue. According to what has been mentioned in this review, due to its antibacterial, anti-inflammatory, and osteo-proliferative activities, QRC and its derivates could represent a valid alternative for the treatment of bacterial infections in the oral cavity and in the rehabilitation of patients having dental bone defects or peri-implantitis. Furthermore, the application of QRC locally by a scaffold and nanocomposite allow to overcome the problem of its low oral bioavailability, which would be useful for the clinical field. Finally, functionalizing the surface of dental implants with this molecule could improve the success rate of the implants after insertion, especially in patients suffering from bone mass loss.

## Figures and Tables

**Table 1:** Summary of in vitro QRC antibacterial activity and bone tissue regeneration properties.

Antibacterial Activity	QRC Concentration	Cell Type	MIC	Markers	Findings	References
	From 250 to 1000 µg/mL	*Staphylococcus aureus; Staphylococcus saprophyticus*	*Staphylococcus aureus* MIC = 62.5 µg/mL; *Staphylococcus saprophyticus* MIC = 1000 µg/mL;		50% reduction of biofilm formation for each bacterium	Dias da Costa Júnior et al. 2018 [40]
	Up to 500 times higher the MICs	*Escherichia coli; Staphylococcus aureus*	*Escherichia coli* MIC = 0.0082 µmol/mL; *Staphylococcus aureus* MIC = 0.0068 µmol/ml		Cell wall and membrane damaged at concentration of 50-fold and 10-fold higher than the *E. coli* and *S. aureus* MICs, respectively.	Wang et al. 2018 [41]
	0.1, 0.05, 0.025 g/mL	*Porphyromonas gingivalis; Aggregatibacter actinomycetemcomitans*	*Porphyromonas gingivalis* MIC = 0.0125 g/mL; *Aggregatibacter actinomycetemcomitans* MIC = 0.1 g/mL		Reduction of bacteria’s growth in time-dependant manner	Geoghegan et al. 2010 [42]
**Osteoprotective Activity**						
	From 0.1 μM to 5 μM	BMSCs		ALP; RUNX2; OXS	Increase of cells proliferation and up regulation of osteogenic genes.	Pang et al. 2018 [50]
	1, 10 and 50 μM	MG-63		ALP; ERK Pathway; ERs	Increase of ALP activity, related to ERK and ERs pathways.	Prouillet et al. 2004 [51]
	0.01, 0.1, 1, 10 and 100 μM	MSCs		ALP, BGP, COL I; MAPK and ERK pathways	Increase of ALP activity and BGP and COL I levels. Increase of ERK and MAPK.	Li et al. 2015 [52]
	Increasing up to 10 μM	RAW 264.7 and PBMC		NFkB; AP-1	Strong inhibition of osteoclast proliferation and differentiation.	Wattel et al. 2004 [53]

ALP: alkaline phosphatase; RUNX2: runt-related transcription factor 2; OXS: Osterix; ERK: extracellular signal-regulated kinases; ER: oestrogen receptor; BGP: osteocalcin; COL I: collagen type 1; MAPK: mitogen-activated protein kinase; NFkB: nuclear factor kappa-light-chain-enhancer of activated B cells; AP-1: activator protein 1.

**Table 2:** Summary of both the in vitro and in vivo QRC-based formulations’ activities on periodontal tissue.

Quercetin-Based Formulation	Dosage	Model of Analysis	Activity Evaluation	Markers	Findings	References
Solution	From 1 to 25 μM	In vitro on HDPs culture	Dentinogenic	ALP, DSPP’s mRNA	Increase of each marker’s expression and mineral deposition in dose-dependent manner	Kim et al. [54]
Solution	6.5% *w*/*v*	Ex vivo demineralized root fragments	Remineralization after artificial injuries		Inhibition of demineralization and promotion of remineralization.	Epasinghe et al. [55]
Composite dental-adhesive	From 100 to 1000 µg/mL	In situ on human third molars; In vitro on *Streptococcus mutans* culture.	Preservation of dentin-adhesive bond strength	MMPs	Reduction of *Streptococcus mutans* metabolic activity and biofilm generation. Reduction of nanoleakage expression and MMPs activity.	Yang et al. [56]
Solution	100 mg/kg for 15 days by subcutaneous route	In vivo on mice infected with *Aggregatibacter actinomycetemcomitans*	Anti-periodontitis and anti-inflammatory	IL-1β, TNF-α, IL-17, I-CAM, RANKL.	Downregulation of cytokines proinflammatory, adhesion molecules and osteoclastogenic genes expression. Reduction of alveolar bone resorption.	Napimoga et al. [57]
Collagen matrix		In vivo on New Zealand white rabbits having parietal bone defects	Bone restoration		Increase of 556% in new bone formation compared to control.	Wong et al. [58]
Hydroxyapatite bioceramic microspheres	200 μM	In vivo on OVX rats with monocortical plug bone defects	Bone restoration		Massive new bone mass and vessel formation after eight weeks from implantation.	Zhou et al. [59]
Composite poly (L-lactide)/chitosan scaffold	200 μM	In vitro on MC3T3-E1 and RAW 264.7 cells culture	Osteogenic and anti-inflammatory	ALP, Runx-2, COL-I, OCN, TNF-α and IL-6	Increase of ALP activity, calcium deposition and osteogenic-related genes expression. Reduction of pro-inflammatory cytokine expression.	Zhu et al. [60]
Hydroxyapatite scaffold	Increasing up to 3.1 wt%	In vitro on OB, OC and ED triculture model	Osteogenic	ALP, Runx-2, COLL1, OPG, RANKL	Increase of OB proliferation and differentiation. Promotion of angiogenetic process. Downregulation of OC differentiation.	Forte et al. [61]
Titanium surface modification	1 mM	In vitro on hGF culture	Anti-inflammatory and tissue-regeneration	Collagens’ mRNA and COX2 mRNA	Stimulation of collagen’s production and reduction of anti-inflammatory processes	Gomez-Florit et al. [74]
Titanium surface modification	1 mM	In vitro on RAW264.7 cell culture In vivo on female New Zealand white rabbits	Anti-resorption activity	Ctsk, H^+^ATPase, Mmp9, RANKL, ALP and LDH	Reduction of osteoclast activity both in vitro and in vivo studies.	Còrdoba et al. [75]

OB: osteoblast; OC: osteoclast; ED: endothelial cells; DSPP: dentin sialophosphoprotein; MMPs: metalloproteinases; IL-1β: interleukin-1β; TNF-α: tumour necrosis factor-α; IL-17: interleukin-17; I-CAM: intercellular adhesion molecules; RANKL: receptor activator of nuclear factor kappa-Β ligand; OPG: osteoprotegerin; COX2: cyclooxygenase type 2; LDH: lactate dehydrogenase.
